# Malaria resurgence after significant reduction by mass drug administration on Ngodhe Island, Kenya

**DOI:** 10.1038/s41598-019-55437-8

**Published:** 2019-12-13

**Authors:** Wataru Kagaya, Jesse Gitaka, Chim W. Chan, James Kongere, Zulkarnain Md Idris, Changsheng Deng, Akira Kaneko

**Affiliations:** 1 Department of Parasitology & Research Center for Infectious Disease Sciences, Graduate School of Medicine, Osaka City University, 1-4-3, Asahimachi, Abeno-ku, Osaka 545-8585 Japan; 2 Department of Clinical Medicine, Mount Kenya University, PO Box 342-01000, Thika, Kenya; 3 Island Malaria Group, Department of Microbiology, Tumor and Cell Biology (MTC), Karolinska Institutet, Biomedicum, Solnavägen 9, 171 65 Solna, Stockholm Sweden; 4 Department of Anthropology, Binghamton University, Binghamton, NY 13905 USA; 5 Nairobi Research Station, Nagasaki University Institute of Tropical Medicine-Kenya Medical Research Institute (NUITM-KEMRI) Project, Institute of Tropical Medicine (NEKKEN), Nagasaki University, PO Box 19993-00202, Nairobi, Kenya; 6 Department of Parasitology and Medical Entomology, Faculty of Medicine, Universiti Kebangsaan Malaysia Medical Centre, 56000 Kuala Lumpur, Malaysia; 7 Science and Technology Park, Guangzhou University of Chinese Medicine, Guangzhou 510006 Guangdong, People’s Republic of China; 8 Institute of Tropical Medicine (NEKKEN), Nagasaki University, Nagasaki, 1-12-4 Sakamoto, Nagasaki, 852-8523 Japan

**Keywords:** Malaria, Epidemiology

## Abstract

Although WHO recommends mass drug administration (MDA) for malaria elimination, further evidence is required for understanding the obstacles for the optimum implementation of MDA. Just before the long rain in 2016, two rounds of MDA with artemisinin/piperaquine (Artequick) and low-dose primaquine were conducted with a 35-day interval for the entire population of Ngodhe Island (~500 inhabitants) in Lake Victoria, Kenya, which is surrounded by areas with moderate and high transmission. With approximately 90% compliance, *Plasmodium* prevalence decreased from 3% to 0% by microscopy and from 10% to 2% by PCR. However, prevalence rebounded to 9% by PCR two months after conclusion of MDA. Besides the remained local transmission, parasite importation caused by human movement likely contributed to the resurgence. Analyses of 419 arrivals to Ngodhe between July 2016 and September 2017 revealed *Plasmodium* prevalence of 4.6% and 16.0% by microscopy and PCR, respectively. Risk factors for infection among arrivals included age (0 to 5 and 11 to 15 years), and travelers from Siaya County, located to the north of Ngodhe Island. Parasite importation caused by human movement is one of major obstacles to sustain malaria elimination, suggesting the importance of cross-regional initiatives together with local vector control.

## Introduction

The estimated global malaria incidence and death decreased by 16% and 48% respectively between 2000 and 2017^1^, as a consequence of increasing global financial aids to support the scale-up of new tools such as artemisinin-based combination therapy (ACT), insecticide treated bed nets (ITNs), and rapid diagnostic test (RDT). In the latest framework for malaria elimination^2^, WHO states that all countries should work towards the ultimate goal of malaria elimination, regardless of their current malaria burden. However spatial heterogeneity in transmission dictates setting-specific strategies to control or eliminate malaria. In low transmission settings, introduction of sensitive molecular methods such as polymerase chain reaction (PCR) to field surveillance reveals that submicroscopic infections contribute substantially to residual malaria transmission^3,4^. Most of these submicroscopic infections are asymptomatic and likely chronic, since the infected hosts do not seek treatment. This observation suggests that elimination will require a mass treatment strategy targeting the whole population (i.e. mass screening and treatment (MSAT) and mass drug administration (MDA)), or the risk population (i.e. focal screening and treatment and focal MDA). As the sensitivity of available field diagnostic methods is less than ideal, MDA is considered a more feasible approach to achieve elimination^5,6^. As same reason of MSAT, identifying and targeting risk group is still not effective in disrupting transmission^6,7^. In 2015, WHO recommended MDA for low-transmission settings^7^, however insufficient evidence from moderate- and high-transmission settings limited the appeal of MDA. Furthermore, only few studies have explicitly evaluated the impact of MDA on submicroscopic infections^8,9^.

**Figure 1:**
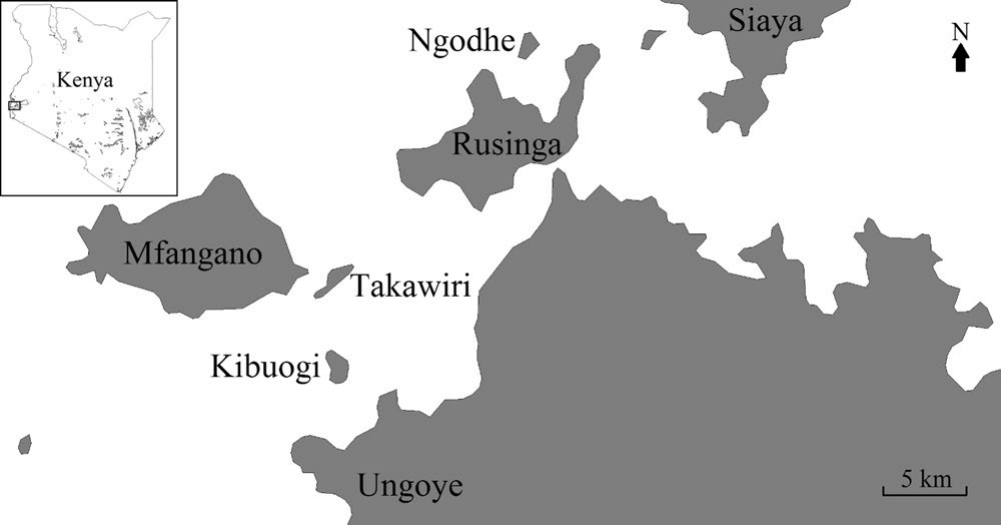
Map of the study site in the Lake Victoria basin. Ngodhe Island is approximately 1 km^2^ in size and 3 km from nearest island, Rusinga, which is connected to the mainland by a bridge. The map was created with DIVA-GIS, version 7.5.0, http://www.diva-gis.org/.

In the Lake Victoria basin of western Kenya, *Plasmodium falciparum* parasite rate for the population aged 2–10 years old (P*f*PR_2–10_) is more than 40%^10^. However, our malariometric surveys in Homa Bay County revealed high local heterogeneity in *Plasmodium* prevalence in this area: highest in the coastal mainland site of Ungoye, followed by the large island of Mfangano and lowest on the three small islands of Ngodhe, Kibuogi, and Takawiri (Fig. 1). Importantly a high proportion of infections were asymptomatic and submicroscopic^11^, rationalizing the use of MDA to reduce malaria transmission towards elimination.

In this study we aimed to assess whether malaria can be eliminated by MDA on a small island in the Lake Victoria basin with heterogeneous transmission. Two rounds of MDA with artemisinin/piperaquine (Artequick) and single low dose of primaquine with a 35-day interval were carried out on the entire population of Ngodhe Island in 2016 before the onset of the long rainy season. Simultaneously provision of ITN was re-strengthened. Subsequent surveys showed that malaria prevalence by microscopy decreased to zero after MDA, however it rebounded to the initial level six months after the completion of MDA. To characterize the nature of imported cases as one of the major causes of resurgence, we further investigated infection status among arrivals at two beaches on Ngodhe for a year.

## Results

### MDA coverage and compliance on Ngodhe

High MDA coverage and compliance were achieved on Ngodhe Island (Table 1). Compliance was approximately 90% in both rounds. The ≥16 years age group had significantly lower compliance (85% and 82% in round 1 and 2, respectively) compared to the other age groups (p < 0.001).

**Table 1:** Coverage and compliance of mass drug administration (MDA) on Ngodhe Island.

	1st round	2nd round
Present on island (n)	486	501
Enrolled (n)*	485	494
Participated (n)^†^	461	460
Completed (n)^‡^	441	438
Coverage (%)^§^	94.9	91.8
Compliance (%)^¶^	90.7	87.4

*Number of residents met by drug administration teams.

^†^Number of residents who took at least one Artequick dose.

^‡^Number of residents who completed all drug treatment.

^§^Number of participated divided by number of present on island.

^¶^Number of completed divided by number of present on island.

### Adverse events

No serious adverse events were reported by MDA participants or observed by the drug administration teams in either MDA rounds on Ngodhe. A total of 37 minor adverse events such as dizziness (10), abdominal discomfort (7), nausea/vomit (6), fatigue (5) and weakness (4) were reported by MDA participants (Table 2). More events were reported in the 1^st^ round (30) than the 2^nd^ round (7). Most of the adverse events (35/37) were reported by participants in the ≥16 years age group.

**Table 2:** Reported adverse events after drug administration on Ngodhe Island (Number of reports).

Adverse event	1st Round	2nd Round	Total
Dizziness	9	1	10
Abdominal discomfort	5	2	7
Nausea/vomit	2	4	6
Fatigue	5	0	5
Weakness	4	0	4
Headache	3	0	3
Feverish	1	0	1
Joint pain	1	0	1

### Parasite prevalence

**Figure 2:**
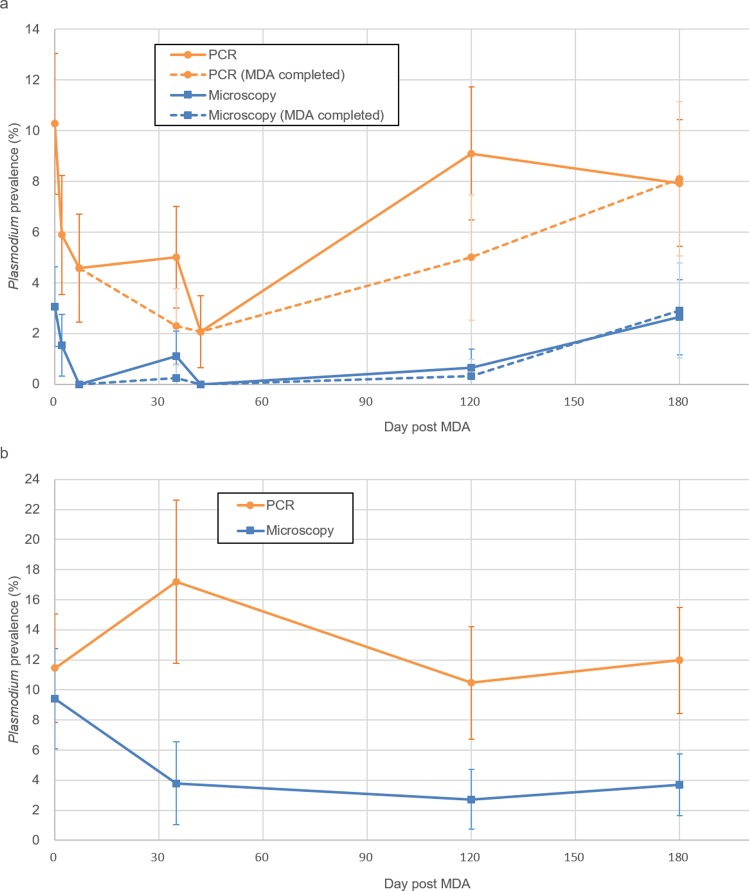
Malaria prevalence by microscopy and PCR after MDA. (**a**) Ngodhe Island, (**b**) Kibuogi Island. Each point corresponds day 0, 2, 7, 35, 42, 120, 180 in (**a**) and day 0, 35, 120, 180 in (**b**) in chronological order.

Changes in microscopy and PCR prevalence over the course of the MDA rounds and follow-up periods on Ngodhe are shown in Fig. 2a. By microscopy, prevalence decreased from 3.1% on day 0 to 1.5% on day 2 and 0% on day 7 (p < 0.001). By PCR, prevalence decreased from 10.3% on day 0 to 5.9% on day 2 and 4.6% on day 7 (p < 0.001). The increase in parasite prevalence between the completion of round 1 (day 7) and the start of round 2 (day 35) was not significant by either microscopy (0% to 1.1%; p = 0.32) or PCR (4.6% to 5.0%; p = 0.11). After drug administration in round 2, prevalence decreased significantly by PCR (5.0% on day 35 to 2.1% on day 42; p = 0.007) but not by microscopy (1.1% to 0%; p = 0.08). In each of the MDA rounds, two individuals who were positive with *P. falciparum* gametocytes by microscopy on the first day were negative seven days after drug administration. Follow-up surveys revealed resurgence in parasite prevalence to levels similar to those before MDA (day 0), 7.9% by PCR (p = 0.29, compared to day 0) and 2.6% by microscopy (p = 0.82) on day 180.

We further categorized positive cases detected on day 35, 120, and 180 by MDA compliance (Fig. 2a). At the start of round 2 (day 35), the majority of positive cases were found in round 1 non-participants; 80% (4/5) by microscopy and 61% (14/23) by PCR. Similarly on day 120, most positive cases were found among non-participants in either MDA rounds; 67% (2/3) by microscopy and 64% (27/42) by PCR. In contrast, on day 180 75% (9/12) and 69% (25/36) of cases detected by microscopy and PCR, respectively, were found in participants who had completed both MDA rounds.

Changes in parasite prevalence on Kibuogi over the same study period are shown in Fig. 2b. Parasite prevalence by microscopy decreased significantly (p = 0.02) from 9.4% (28/297) on day 0 to 3.8% (7/185) on day 35, and remained at 2.7% (7/258) and 3.7% (12/324) on days 120 and 180, respectively. Parasite prevalence by PCR did not significantly change over the study period.

Between islands, PCR prevalence did not differ significantly prior to interventions and during the follow-up period, except that on day 35 PCR prevalence on Ngodhe (5.0%) was significantly lower (p < 0.01) than that on Kibuogi (17%).

### Parasite clearance by artequick

We evaluated parasite clearance by Artequick based on the infection status on day 0, 2, 7 and 35 on Ngodhe. By microscopy, 11 of the 14 positive individuals on day 0 completed the two-day Artequick treatment. All of them became negative by day 2 except for one case whereby parasites were cleared by day 7. They remained negative on day 35 except for 3 cases who were absent for round 2 of MDA. Therefore, parasite clearance on day 2, 7 and 35 were 91%, 100% and 100%, respectively. Of these 14 individuals, 5 of them were positive by PCR on day 2, but only one positive by PCR on day 35. This positive case stayed positive by PCR at all sampling points between day 0 to 35.

Of 38 submicroscopic infections detected by PCR on day 0, 34 completed the Artequick treatment. Among those captured during the follow-up, the numbers of positive individuals on day 2, 7, and 35 were 11/32, 6/29, and 2/31, respectively. Therefore, parasite clearance for submicroscopic infection at each time point were 66%, 79% and 94%, respectively.

### Anemia and changes in hemoglobin (Hb) levels after primaquine administration

Three repeated Hb readings (on day 0, 2, and 7) were available from 323 MDA participants who had taken a single low-dose primaquine. Overall anemia prevalence on Ngodhe was 35% (113/323) on day 0 before drug administration. Among them, 46, 63 and 4 individuals were considered mildly, moderately and severely anemic, respectively according to the WHO standards^12^. Changes in mean Hb levels are shown in Table 3. For the overall population, mean Hb showed a slight decrease but there were no significant (p = 0.201) differences among the three time points. However, a significant (p < 0.001) decrease in mean Hb was observed for those who were non-anemic on day 0. Post hoc tests with the Bonferroni correction showed that the reduction was significant from day 0 to day 2 (p < 0.001), but not from day 2 to day 7 (p = 1.00), suggesting that the reduction in Hb level by primaquine was short-lived. We observed a significant increase (p = 0.001) in mean Hb levels after primaquine administration among those who were moderately to severely anemic on day 0. However, of the 17 individuals in this group who showed ≥10% increase in Hb level between days 0 and 2, their Hb levels decreased by a mean of only 3.6% between days 7 and 2, suggesting that inaccurate measurement on days 0 likely resulted in erroneously low reading.

**Table 3:** Mean (SD) hemoglobin levels in g/dl after single low-dose primaquine administration by anemia status.

	Day 0	Day 2	Day 7	p*
Moderate/severe (n = 67)	9.68 (1.08)	10.12 (1.83)	10.23 (1.89)	0.001
Mild (n = 46)	11.48 (0.73)	11.55 (1.26)	11.79 (1.58)	0.192
Non-anemic (n = 210)	13.72 (1.39)	13.38 (1.64)	13.32 (1.69)	<0.001
Overall (n = 323)	12.56 (2.09)	12.45 (2.11)	12.46 (2.13)	0.201

*Repeated measures analysis of variance.

We also evaluated the relative changes in Hb individually. Of 323 study participants, only four (1.2%) showed reductions in Hb levels on day 2 to less than 80% of their respective levels on day 0 (range: 63–80%). By day 7, Hb levels in these four individuals had recovered, reaching 81% to 98% of their respective levels on day 0.

### Arrivals on Ngodhe Island

From June 2016 to September 2017, 419 individuals who arrived at Ngodhe by boats provided samples for determination of infection status (Table 4). Among these arrivals, 4.6% and 16% were positive by microscopy and PCR respectively, and 88% of PCR positive cases were submicroscopic infection. Multivariate logistic regression analysis revealed the following factors significantly associated with malaria infections by PCR: age groups 0–5 years (p = 0.003) and 11–15 years (p < 0.001), and arrivals from Siaya County (p < 0.001) (Table 5). We did not find any significant association between the purpose of visiting Ngodhe and malaria infection.

**Table 4:** The characteristics of the arrivals on Ngodhe Island (N = 419).

Variable		Number	%
Gender	Male	176	42
	Female	243	58
Age group in years	0–5	37	8.8
	6–10	35	8.4
	11–15	65	16
	16–30	182	43
	≥31	100	24
Resident/Non-resident	Resident	179	44
	Non-resident	225	56
Purpose of visit Ngodhe (Non-resident)	School holiday	77	34
	Visiting friends/relatives	54	24
	Fishing	38	17
	Business	29	13
	Others	27	12
Point of departure	Homa Bay	175	61
	Siaya	76	26
	Migori	21	7.3
	Other counties/nations	15	5.2

**Table 5:** Risk factors for malaria infection among arrivals.

Variable		Unadjusted OR	95% CI	p	Adjusted OR	95% CI	p
Gender	Male	1.11	0.65–1.87	0.710			
	Female	1 (ref)					
Age group in years	0–5	5.26	1.59–17.36	0.006	7.63	2.01–28.98	0.003
	6–10	6.37	1.96–20.66	0.002	3.91	0.95–16.13	0.059
	11–15	6.52	2.27–18.74	0.001	9.24	2.72–31.38	<0.001
	16–30	3.41	1.27–9.15	0.015	3.28	1.15–9.35	0.026
	≥31	1 (ref)			1 (ref)		
Resident/Non-resident	Resident	1 (ref)					
	Non-resident	1.45	0.84–2.51	0.182			
Point of departure	Homa Bay	1 (ref)			1 (ref)		
	Siaya	4.35	2.16–8.74	<0.001	4.96	2.36–10.41	<0.001
	Migori	2.83	0.92–8.70	0.069	2.27	0.71–7.30	0.168
	Other counties/nations	2.27	0.58–8.83	0.239	2.92	0.69–12.38	0.145

## Discussion

Two rounds of MDA with artemisinin/piperaquine and primaquine led to a significant reduction in *P. falciparum* prevalence on Ngodhe Island in Lake Victoria, Kenya, although the impact was short-lived. Since the island population was small and parasite prevalence on day 0 was low (3.1% by microscopy and 10.3% by PCR), this study had a low power to detect statistically significant differences. We believe that MDA is an important tool to overcome the problem of widespread submicroscopic infections. On Aneityum Island in Vanuatu, the authors demonstrated that malaria could be eliminated using a short-term MDA and sustained vector control with high degree of local community participation^13^. Further, continuous use of ITNs and a robust community-directed case surveillance system have kept the island malaria free for 25 years, except for a small outbreak of *P. vivax* in 2002^14,15^. Successful malaria elimination by MDA has also been documented on other islands such as Lanyu in Taiwan and Nissan in Papua New Guinea, as well as in relatively isolated villages in Asia^16–18^. Recently large-scale MDA trials were conducted in the Greater Mekong Subregion (GMS) together with increased access to early diagnosis and treatment, resulting in dramatic reduction of malaria incidence albeit for a short term^8,19^. In Africa, recent MDA have shown variable degrees of success in reducing malaria burden and interrupting transmission^6,9,20–22^.

In our study, we evaluated malaria prevalence before and after MDA on Ngodhe by both microscopy and PCR. PCR allowed for detection of infections that went undetected by microscopy seven days after treatment in both MDA rounds. Parasite resurgence was also detected earlier by PCR (day 120) than microscopy (day 180). Our results clearly indicate that evaluations based on microscopy alone likely overestimate the impact of MDA and underestimate the risk of resurgence after MDA. This trend is more pronounced in case of MSAT, which screens the target population using RDT and treats only positive cases. Since RDTs have similar sensitivity as microscopy and often fail to detect low-density i.e. submicroscopic infections, the impact of MSAT on interrupting transmission is limited^23^. Our follow-up on Kibuogi Island was similar to MSAT except that treatment was not directly observed. We observed no significant impact of MSAT on PCR prevalence on Kibuogi, while MDA on Ngodhe did (Fig. 2). On Ngodhe, by day 35 MDA had cleared 32 submicroscopic infections which might not have been detected by MSAT. While residual PCR positivity could be a result of slow clearance of parasite debris from circulation similar to that in sleeping sickness or brucellosis^24^, viability and transmissibility of malaria parasites of the residual PCR positivity has been demonstrated in a study with rodent malaria^25^. Furthermore, the transmissibility of submicroscopic infections has also been shown in various transmission settings^3,4,26^. A recent report showing the persistence and oscillations of submicroscopic malaria infections in Vietnam also suggests the importance of following up submicroscopic infections^27^. To the best of our knowledge, few previous reports showed the impact of MDA on parasite prevalence including submicroscopic infections^8,28,29^. The study in the GMS showed significant reduction to 0.4% *P. falciparum* prevalence by ultra-sensitive PCR three months after MDA. The more pronounced impact observed in that study when compared to ours may be attributed to the different drug regimens (three rounds of three daily doses of dihydroartemisinin-piperaquine in the GMS) and lower initial prevalence. On the other hand, current PCR-based evaluation require laboratory infrastructure, skilled technicians, and are costly, making their application in peripheral settings impracticable. Loop-mediated isothermal amplification (LAMP) requires fewer skills and less infrastructure but is still costly. Recently high-sensitivity RDTs have become available, though their diagnostic sensitivity is still lower than conventional PCR^30,31^; thus the development of inexpensive, sensitive, and field-applicable alternatives to PCR is still highly desired.

Compared to previous MDA that used the artemisinin/piperaquine combination (Artequick), our study showed similar efficacy in clearing parasite infections. In Cambodia, MDA with same dose of Artequick as that in our study plus 9 mg of primaquine reduced parasite rates by microscopy from 52% to 2.6%^18^. A similar MDA schedule implemented in the Comoros (population 300,000) reduced monthly malaria morbidity by 99% for one year and now the country is on track to achieve national malaria elimination^22^. The two-day artemisinin/piperaquine regimen may have some advantages over other ACTs that require a three-day treatment course such as dihydroartemisinin/piperaquine. Piperaquine is known to cause QT prolongation^32^ and the risk of cardiotoxicity appears to correlate with a total dose of piperaquine^33^. In this study, the total dose of piperaquine administered to adults was 1500 mg (750 mg/day for two days), compared to 2880 mg (960 mg/day for three days) in the MDA using dihydroartemisinin/piperaquine^6,19^. The lower piperaquine dose in our MDA schedule likely provided greater safety margins without compromising overall efficacy. In addition, the two-day regimen had obvious advantages over three-day regimen to achieve the higher MDA compliance as discussed below. However, it should be noted that in our study one person remained positive by PCR on day 35 despite completion of Artequick treatment. It is unclear if a higher total dose of piperaquine as contained in the dihydroartemisinin/piperaquine combination would have cleared that infection.

High MDA compliance (around 90%) on Ngodhe was mainly owed to robust community engagement through frequent meetings, discussion, feedback sessions and active involvement of community health volunteers. Since children under 15 years were mainly captured in school, they had higher compliance compared with the adults. A shorter duration (two days) of drug administration also reduced the workload of the drug administration teams. A review of previous MDA studies concludes that high MDA coverage (at least 80% or even 90%) is one of the primary factors determining the success of MDA^34^, as the promised benefits to the community can be annulled by non-participation of a small number of individuals^35^. Our previous study in Vanuatu showed that complaints of minor side effects could be one of the factors which reduce MDA compliance^13^. In this MDA, with the exception of a few individuals (<15) who were provided with the second Artequick dose with instructions due to their imminent departure from the island, all drug intakes were directly observed by a nurse. Furthermore, side effects were monitored with the assistance from community volunteers in drug administration team. On the other hand, MDA trials in the Gambia and Myanmar identified unstable and/or seasonal population movement as another barrier for achieving high compliance^36,37^.

One of the issues previously hampering the recommendation of MDA was the possibility of accelerating the selection of drug resistant parasites^38,39^. The risk of promoting drug resistance is however thought to be low, since MDA is most impactful in clearing parasites among submicroscopic carriers rather than in patients with levels of parasitemia that are often over a thousand times higher, where there may be a greater chance of de novo emergence of resistant parasites^40,41^. Another concern is that poor adherence might lead to underdosing, resulting in drug concentrations which are too low to clear all parasites but are high enough to select for drug tolerance. Thus, directly observed treatment and close monitoring of adverse events (e.g. vomiting within an hour of drug administration) will be critical to prevent the selection and spread of drug resistant parasites. Recent large-scale MDA trials in eastern Myanmar and the Comoros reported no evidence for selection of artemisinin resistant molecular markers after MDA^19,22^.

Although primaquine was known to potentially cause hemolysis in glucose-6-phosphate dehydrogenase (G6PD) deficient individuals^42^, after a thorough review of available evidence WHO now recommends 0.25 mg base/kg primaquine as a safe and effective dose against *P. falciparum* gametocytes. In our study site, G6PD deficiency is observed in about 10% of males, most of whom carry the G6PD A- allele (unpublished data). We administered a 0.13 mg base/kg primaquine dose on the first day of treatment and observed no hemolytic sign from any participants and negligible changes in hemoglobin levels. Together with the MDA in Cambodia, which employed 0.15 mg base/kg primaquine dose and reported a reduction in *P. falciparum* gametocyte prevalence from 13% to 0.8%^18^, our study further confirms the safety and the gametocytocidal effectiveness of this lower than recommended primaquine dose. The effectiveness of the lower primaquine dose is also supported by *in vitro* oocyst and sporozoite formation test^43^.

Analysis of parasite prevalence by PCR after MDA on Ngodhe indicated that despite high compliance, MDA did not completely eliminate the parasite reservoir on the island. The remaining submicroscopic infections after MDA, combined with local residual vector capacity, likely represented a source of parasite resurgence.

The resurgence after significant reduction in parasite prevalence by MDA also highlighted the importance of controlling vulnerability (parasite importation by human movement) and receptivity (transmission by local vector) in the context of sustainable malaria elimination^44^. Since parasite prevalence on Kibuogi Island did not increase significantly over the same period, it is difficult to attribute the increase in prevalence on Ngodhe Island to seasonality or rainfall pattern. Furthermore detailed analysis in the follow-up period after MDA revealed that more than half of the PCR-positive cases detected on days 35 and 120 were found among visitors or island residents returning from other areas, indicating parasite importation to the island. On the other hand, most PCR-positive cases on day 180 were found in MDA participants, indicating that the prophylactic impact of MDA had vanished by this time. In contrast to Aneityum Island in Vanuatu, where a high degree of geographic isolation facilitated the long term sustainability of malaria elimination^13^, our study site in Lake Victoria features a significant degree of human movement. During our study, the numbers of enrolled populations on Ngodhe fluctuated between 463 and 494, suggesting a very mobile population. Our surveillance on the arrivals at the Ngodhe beaches identified several risk groups, such as arrivals from Siaya County where malaria prevalence is higher. Ngodhe lies close to the water border between Kenya and Uganda, and our surveillance identified a number of visitors from other counties and countries, highlighting the importance of cross-regional initiatives to minimize the risks of parasite importation especially in the periphery of the administrative areas. A recent MDA study in the GMS experienced resurgence following very low prevalence by ultra-sensitive PCR, and concluded that importation of malaria infection as a factor which undermined the impact of MDA^8^. The same conclusion was reached after a MDA trial in Zanzibar, which is geographically separated from the Tanzanian mainland, reflecting very high population mobility in Africa even in island settings^9^. Recently, spatio-temporal modeling approach has been featured in the context of defining local heterogeneity in transmission intensity or hotspots^45,46^. More detailed information about the extent and pattern of human movement will help us understand its contribution to malaria transmission in the study area.

In light of the difficulty in preventing parasite importation by human movement and the fact that imported parasites are transmitted by local residual mosquitoes, innovative methods to limit local transmission by *Anopheles* mosquitoes are crucial to limit resurgence after MDA. On Ngodhe, ITN coverage was strengthened through distribution of free nets to replace damaged or missing nets as part of the intervention package, and self-reported ITN use rose from 62% in August 2015 to 71% in May 2016 (day 120), although the latter figure may still be too low to effectively interrupt local transmission. ITN use among school children is particularly low because many of them sleep on the floor^47^. Recently over-allocation of ITNs has also become a subject of discussion^48^, and on Ngodhe we witnessed many instances of ITN misuse, indicating human behavioral modification as an important factor to enhance effectiveness of vector control measures. Relevant community programs for proper use of ITNs are required. ITNs specifically tailored to cover the open space between the roof and the wall in typical residential structures (“ceiling nets”) can provide additional protection against exposure to vectors^49,50^. Installation of ceiling nets with ITN material in the study area are currently under consideration.

As in other malaria-endemic areas of tropical Africa, the mass distribution of long lasting insecticide treated bed nets (LLINs) in the Lake Victoria basin has produced pyrethroid resistant mosquitoes and shifted the predominant vector species from *Anopheles gambiae* to *Anopheles arabiensis*^51,52^, which is relatively exophagic in feeding behavior. These developments may drastically reduce the power of existing vector control tools such as LLINs and indoor residual spray. Although new classes of insecticides such as chlorfenapyr and piperonyl butoxide (PBO) are being investigated or incorporated into existing products, they do not overcome vector behavioural resistance. In these circumstances, ivermectin, a drug that has been extensively and safely used for onchocerciasis and lymphatic filariasis since the 1980s, has emerged as a novel drug-based vector control tool to reduce the lifespan of *Anopheles* mosquitoes^53,54^, and could be included as a partner to ACTs in MDA for malaria elimination.

In conclusion, high MDA compliance temporarily suppressed both microscopic and submicroscopic *Plasmodium* infections on Ngodhe Island. However, due to the island’s proximity to areas with moderate and high transmission, suppression of parasite importation and local vector capacity is critical for sustainable elimination. This study illustrates the challenges of using MDA to locally eliminate malaria in a heterogeneous transmission setting, which represents many malaria endemic areas in tropical Africa.

## Methods

### Ethics approval and consent to participate

The study was approved by the Kenyatta National Hospital/University of Nairobi-Ethics and Research Committee in Kenya (registration number: P609/10/2014), and performed in accordance with relevant guidelines and regulations. Community consent to study participation was sought through workshops and sensitization meetings with the island communities. Informed consent was obtained from all study participants at enrollment.

### Description of the study site

Two rainy seasons are observed in the Lake Victoria basin: the long rainy season runs from March to June and the short rainy season from November to December. Malaria incidence peaks one to two months after the rainy seasons. *Anopheles gambiae s.s*. is the predominant vector on islands in Lake Victoria^51^.

The islands of Ngodhe and Kibuogi in Homa Bay County, Kenya were chosen for this study due to their similarities in physical geography, population, and malaria transmission intensity (Table 6)^55^. A sample size calculation suggested that the population of islands would provide 80% power to detect a 50% reduction of prevalence from a 10% prevalence by PCR. Ngodhe is a small island (<1 km^2^) located 3 km to the north of Rusinga Island, which is connected to the town of Mbita via a bridge. Kibuogi (1.5 km^2^) is situated approximately halfway between the island of Mfangano and the mainland coast. Subsistent horticulture, animal husbandry, and small-scale commercial fishing are the major economic activities on these islands. Access to these two islands is restricted to small boats. Previous malariometric surveys revealed similar level of malaria prevalence and *P. falciparum* predominance on Ngodhe and Kibuogi (Table 6)^11,55^. LLINs were distributed for free by the Ministry of Health on both islands prior to our study. The increases in ITN use between 2012 and 2015 were accompanied by the decrease in malaria prevalence similarly on both islands (Additional file 1). Concurrent with the MDA (January to March 2016), additional LLINs were distributed for free to households with missing and/or damaged nets on both Ngodhe and Kibuogi by the study team.

**Table 6:** Baseline data of Ngodhe and Kibuogi Islands.

	Ngodhe	Kibuogi
Land area (km^2^)*	0.98	1.54
Households^†^	137	108
Population^†^	563	502
Median age (IQR)^†^	18 (8–30)	15 (7–28)
% male (95% CI)^†^	51 (47–55)	50 (46–55)
Microscopy parasite rate (95% CI)^‡^	6.6 (5.4–7.9)	6.3 (4.9–7.8)
PCR parasite rate (95% CI)^‡^	16 (14–18)	16 (14–18)
Geometric mean of parasite density (parasite/µl blood)^‡^	1021	1720
% reported bed net use (95% CI)^§^	49 (46–52)	44 (41–48)

^*^Estimated using, https://www.daftlogic.com/projects-google-maps-area-calculator-tool.htm.

^†^Nagasaki University-Health and Demographic Surveillance System (HDSS).

^‡^Data from 2012 to 2015^9^.

^§^Data from 2013 to 2015.

### MDA on Ngodhe

The MDA protocol on Ngodhe was adopted from the protocol used by Prof. Li Guoqiao in Cambodia^18^ with minor modifications. Two rounds of MDA covering the entire population on Ngodhe Island were conducted with a 35-day interval between January and March 2016, before the onset of the long rainy season.

The MDA schedule of the 1^st^ round was: day 0 - two tablets of 62.5 mg artemisinin plus 375 mg piperaquine combination (Artequick®) along with 8 mg primaquine; day 1 - two tablets of Artequick. Doses for children were adjusted as fractions of the adult dose based on age: one-eighth (<1 years), one-fourth (1–3 years), three-eighths (4–6 years), one-half (7–10 years), and three-fourths (11–15 years). Specially formulated primaquine tablets of 8 mg and 2 mg were kindly provided by Prof. Li. Pregnant women in their first trimester were given piperaquine over three consecutive days (600 mg each on day 0 and 1, and 300 mg on day 2) in lieu of Artequick, and 8 mg primaquine on day 0. The same schedule was repeated in the 2^nd^ round (day 35 and 36).

**Figure 3:**
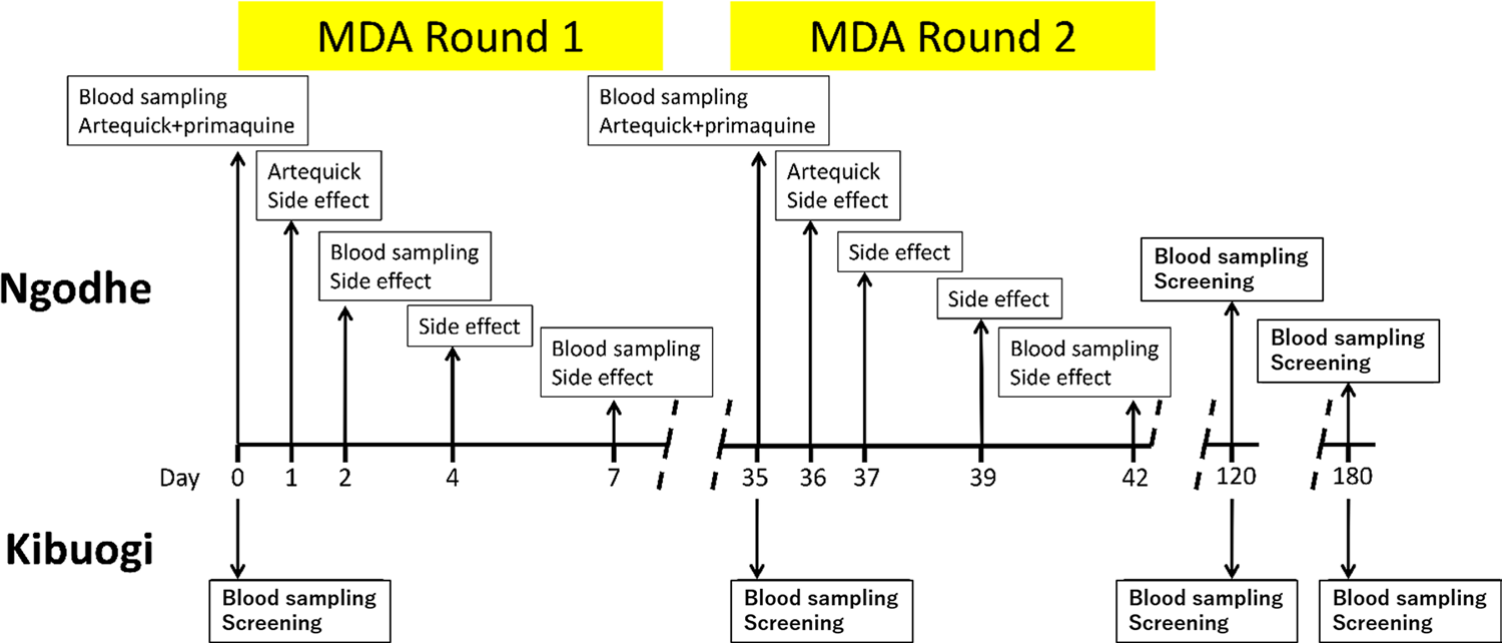
MDA and follow-up schedule.

Ngodhe Island was divided into three areas, each of which consisted of approximately 45–50 households. All households in each area were visited by a drug administration team consisting of two village health volunteers, two laboratory technicians, and one nurse. For individuals who were absent during the initial visit, multiple attempts at various times (at least two: one in the morning and one in the afternoon and/or evening) of the day were made over the subsequent four days to maximize MDA coverage and compliance. Drugs were administered under direct supervision of the team except for a small number of instances (<15) where participants intending to depart the island were provided the second artemisinin-piperaquine dose with instructions. Side effects and adverse events were assessed by open-ended questions during follow-up house visits on day 1, 2, 4, and 7 (Fig. 3).

All permanent and temporary residents on Ngodhe were eligible to participate in MDA. Visitors who did not spend any night on Ngodhe during the MDA rounds were not included. Participants under artemether-lumefantrine treatment (7 individuals in 1^st^ round) received only primaquine on day 0. Eligible individuals with Hb levels below 7 g/dl on day 0 (before drug administration) were not given primaquine, and those with complicated or severe malaria and/or other serious illnesses (e.g. impaired consciousness, respiratory distress, convulsions, and abnormal bleeding) were excluded from MDA. Hb levels on day 0, 2, and 7 were measured with the HemoCue 201 + System (HemoCue AB, Ängelholm, Sweden).

### Parasite prevalence

On Ngodhe, *Plasmodium* infection status was determined on day 0, 2, 7, 35, 42, 120, and 180. Blood sampling on day 0 and 35 took place before drug administration. On Kibuogi, infection status was determined on day 0, 35, 120, and 180. The surveys were conducted by house visits; a second attempt was made when the residents were found absent during the first visit.

Parasite prevalence was determined by microscopy and PCR. Thick and thin blood smears were prepared on site and transported to the main laboratory in Mbita, where thin smears were fixed with methanol, and all smears were stained with 3% Giemsa solution for 30 minutes and examined by experienced microscopists as previously described^11^. Blood samples (70 µl) were also spotted on Whatman ET31 Chr filter papers (Whatman International, Maidstone, UK) with 75-mm micro-hematocrit capillary tube (Thermo Fisher Scientific, MA, USA), allowed to dry at ambient temperature, and stored in individual zipped plastic bags at −20 °C. DNA was extracted from each of quartered blood spots (17.5 µl) using the QIAamp Blood Mini Kit (Qiagen, Hilden, Germany) according to the manufacturer’s instructions. PCR amplification of the *Plasmodium* mitochondrial cytochrome c oxidase III (*cox3*) gene was performed as described previously^56^. This PCR can differentiate the four major species (*P. falciparum*, *Plasmodium vivax*, *Plasmodium ovale*, *Plasmodium malariae*) and the numbers of infections by each species are shown in additional file 2.

In each prevalence survey on Kibuogi and the day 120 and 180 surveys on Ngodhe, *P. falciparum* infections were also examined on sites using the Paracheck-Pf RDT (Tulip Group, India). Individuals with positive results were treated with a standard course of artemether-lumefantrine in accordance with the guideline of the Ministry of Health in Kenya.

### Surveillance of arrivals on Ngodhe

Surveillance was conducted from June 2016, 3 months post the last MDA round, to September 2017. Boat passengers arriving at the two entry beaches of Ngodhe Island were asked to provide blood samples for detection of *Plasmodium* infections by RDT (Paracheck-Pf; Tulip Group, India), microscopy, and PCR (described above). Visitors with positive diagnosis by RDT were treated with artemether-lumefantrine. Age, sex, purpose of visit, place of departure, and length of stay were recorded for each consenting visitor.

### Data analysis

For each MDA round on Ngodhe, “present on island” and “enrolled” are defined as the true population at each time point and the population captured by drug distribution teams, respectively, as shown in Table 1. Thus those who were away for a few weeks over both rounds of the MDA are included in “present on island”, but not “enrolled”. Coverage and compliance were calculated as “participated” divided by “present on island” and “completed drug administration” divided by “present on island”, respectively.

Parasite prevalence at different time points was compared using the McNemar’s test. Anemia status of MDA participants on Ngodhe was determined based on Hb level on day 0 according to WHO standards^12^ after altitude adjustments. Changes in Hb levels after primaquine administration were examined using the repeated measures analysis of variance (ANOVA).

Logistic regression analysis was performed to identify risk factors associated with positive *Plasmodium* infection as determined by PCR among the arrivals on Ngodhe. Variables included sex, age group, and place of departure. All significant variables (p < 0.05) from the likelihood ratio test in the univariate analysis were entered into a multivariate logistic regression model. All statistical analyses were performed using SPSS v24 (IBM Corporation, Armonk, USA).

## Supplementary information

Supplementary information

## Data Availability

The datasets used and/or analyzed during the current study are available from the corresponding author on reasonable request.
