# Supplementary material for: Malaria resurgence after significant reduction by mass drug administration on Ngodhe Island, Kenya
Source: Sci Rep. 2019 Dec 13;9:19060. doi: 10.1038/s41598-019-55437-8 (PMC6910941; doi:10.1038/s41598-019-55437-8)
Supplement: Supplementary file 1 — Supplementary information [file 41598_2019_55437_MOESM1_ESM.pdf]

Title

Malaria resurgence after significant reduction by mass drug administration on Ngodhe Island, Kenya

Authors

Wataru Kagaya[1#], Jesse Gitaka[2#], Chim W. Chan[1,3,4], James Kongere[5], Zulkarnain Md Idris[3,6], Changsheng Deng[7], Akira Kaneko[1,3,8*]

[1]Department of Parasitology & Research Center for Infectious Disease Sciences, Graduate School of Medicine, Osaka City University, 1-4-3, Asahimachi, Abeno-ku, Osaka, 545-8585, Japan

[2]Department of Clinical Medicine, Mount Kenya University, PO Box 342-01000, Thika, Kenya

[3]Island Malaria Group, Department of Microbiology, Tumor and Cell Biology (MTC), Karolinska Institutet, Biomedicum, Solnavägen 9, 171 65 Solna, Stockholm, Sweden

[4]Department of Anthropology, Binghamton University, Binghamton, NY 13905, USA

[5]Nairobi Research Station, Nagasaki University Institute of Tropical Medicine-Kenya Medical Research Institute (NUITM-KEMRI) Project, Institute of Tropical Medicine (NEKKEN), Nagasaki University, PO Box 19993-00202, Nairobi, Kenya

[6]Department of Parasitology and Medical Entomology, Faculty of Medicine, Universiti Kebangsaan Malaysia Medical Centre, 56000, Kuala Lumpur, Malaysia

[7]Science and Technology Park, Guangzhou University of Chinese Medicine, Guangzhou, 510006, Guangdong, People's Republic of China

[8]Institute of Tropical Medicine (NEKKEN), Nagasaki University, Nagasaki, 1-12-4 Sakamoto, Nagasaki, 852-8523, Japan

[#]These first authors contributed equally to this article

[*]Corresponding author (e-mail: akira.kaneko@ki.se)

Additional file 1 Malaria prevalence by microscopy and PCR, and ITN usage before MDA on Ngodhe Island (A) and Kibuogi Island (B).

A

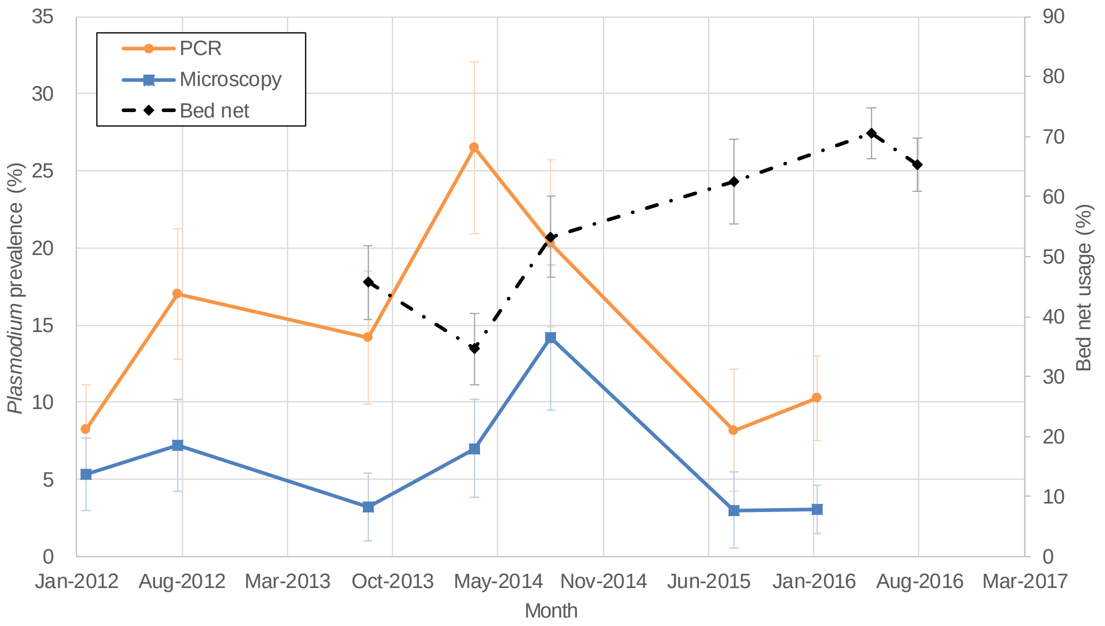

B

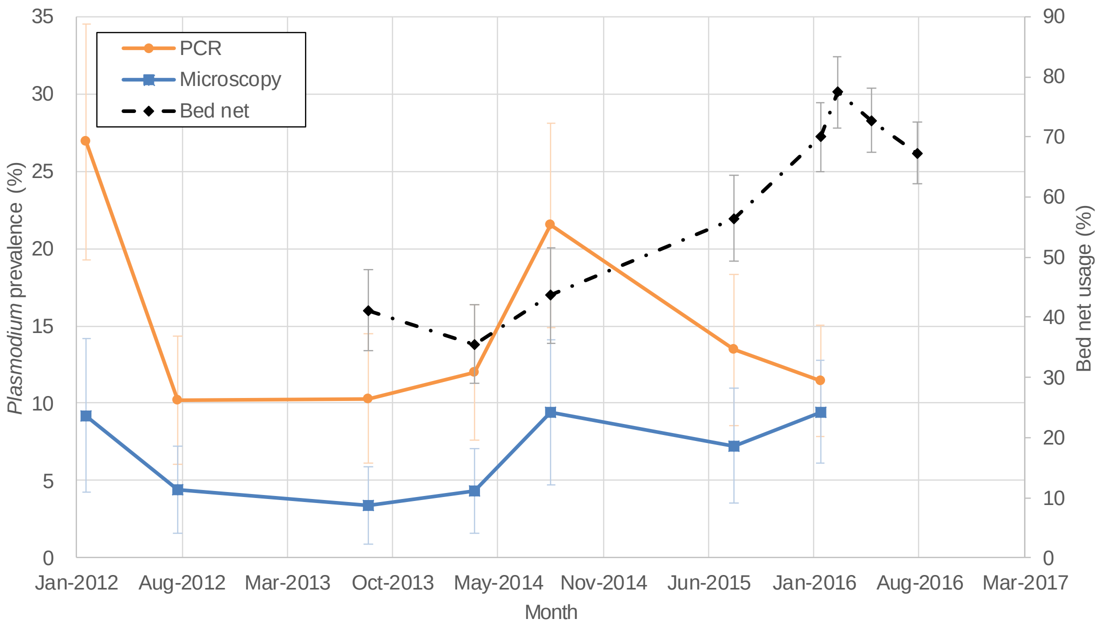

Data between 2012 and 2014 were adapted from [11].

Additional file 2 The number of *Plasmodium* infections by species as detected by PCR during and after MDA on Ngodhe Island (A) and MSAT on Kibuogi Island (B). The number in Parentheses indicates the number of examined samples.

(A)

	Day 0 (458)	Day 2 (391)	Day 7 (372)	Day 35 (459)	Day 42 (387)	Day 120 (462)	Day 180 (454)
P. falciparum	37	19	14	21	8	28	31
P. malariae	1	2	1	0	0	3	1
P. ovale	2	0	0	1	0	3	2
P. falciparum and *P. malariae*	6	1	2	0	0	6	2
P. falciparum and *P. ovale*	1	0	0	0	0	0	0
P. malariae and *P. ovale*	0	0	0	0	0	1	0
P. falciparum, P. malariae and *P. ovale*	0	1	0	1	0	1	0

(B)

	Day 0 (297)	Day 35 (186)	Day 120 (258)	Day 180 (326)
P. falciparum	28	30	23	30
P. malariae	2	1	0	1
P. ovale	0	0	2	3
P. falciparum and *P. malariae*	3	0	2	3
P. falciparum and *P. ovale*	0	0	0	2
P. malariae and *P. ovale*	0	1	0	0
P. falciparum, P. malariae and *P. ovale*	1	0	0	0
